# A Method to Experimentally Estimate the Conductivity of Chronic Stroke Lesions: A Tool to Individualize Transcranial Electric Stimulation

**DOI:** 10.3389/fnhum.2021.738200

**Published:** 2021-10-12

**Authors:** Joris van der Cruijsen, Maria Carla Piastra, Ruud W. Selles, Thom F. Oostendorp

**Affiliations:** ^1^Department of Rehabilitation Medicine, Erasmus MC, University Medical Center Rotterdam, Rotterdam, Netherlands; ^2^Department of Biomechanical Engineering, Delft University of Technology, Delft, Netherlands; ^3^Donders Institute for Brain, Cognition and Behavior, Radboud University Medical Center, Nijmegen, Netherlands; ^4^Department of Plastic and Reconstructive Surgery and Hand Surgery, Erasmus MC, University Medical Center Rotterdam, Rotterdam, Netherlands

**Keywords:** bioimpedance, conductivity measurement, electroencephalography, tDCS, stroke lesion

## Abstract

The inconsistent response to transcranial electric stimulation in the stroke population is attributed to, among other factors, unknown effects of stroke lesion conductivity on stimulation strength at the targeted brain areas. Volume conduction models are promising tools to determine optimal stimulation settings. However, stroke lesion conductivity is often not considered in these models as a source of inter-subject variability. The goal of this study is to propose a method that combines MRI, EEG, and transcranial stimulation to estimate the conductivity of cortical stroke lesions experimentally. In this simulation study, lesion conductivity was estimated from scalp potentials during transcranial electric stimulation in 12 chronic stroke patients. To do so, first, we determined the stimulation configuration where scalp potentials are maximally affected by the lesion. Then, we calculated scalp potentials in a model with a fixed lesion conductivity and a model with a randomly assigned conductivity. To estimate the lesion conductivity, we minimized the error between the two models by varying the conductivity in the second model. Finally, to reflect realistic experimental conditions, we test the effect rotation of measurement electrode orientation and the effect of the number of electrodes used. We found that the algorithm converged to the correct lesion conductivity value when noise on the electrode positions was absent for all lesions. Conductivity estimation error was below 5% with realistic electrode coregistration errors of 0.1° for lesions larger than 50 ml. Higher lesion conductivities and lesion volumes were associated with smaller estimation errors. In conclusion, this method can experimentally estimate stroke lesion conductivity, improving the accuracy of volume conductor models of stroke patients and potentially leading to more effective transcranial electric stimulation configurations for this population.

## Introduction

Non-invasive electric brain stimulation techniques, such as transcranial direct current, alternating current, and random noise stimulation (tDCS, tACS, and tRNS), have been proposed to increase the effectiveness of stroke rehabilitation by passing a small current through the cortical regions related to impaired physiological systems (Schlaug et al., 2008). Although favorable results of non-invasive brain stimulation on stroke survivors have been reported (Kim et al., 2010), systematic reviews indicate that the effectiveness of brain stimulation is not consistent in, among others, motor recovery (Lefebvre and Liew, 2017) and aphasia (Elsner et al., 2019).

A possible cause for the lack of consistent effects is that the electrode configurations used may not lead to stimulation reaching the targeted region as intended (Vöröslakos et al., 2018; Laakso et al., 2019). This effect is even more accentuated in stroke subjects due to the influence of brain lesions on the electric field distribution (Minjoli et al., 2017; Piastra et al., 2021a). Simulation of brain stimulation using MRI-based volume conduction models is a means to quantify and optimize stimulation strength at targeted brain regions and has been applied in both healthy subjects (Wagner et al., 2007) and many patient populations, including stroke subjects (Wagner et al., 2007; Datta et al., 2011).

**FIGURE 1:**
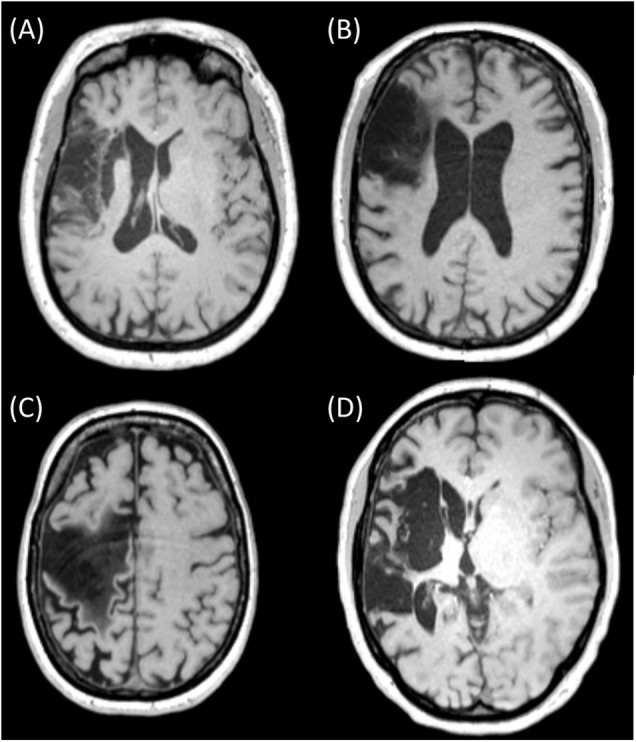
MRI slices of four different chronic stroke subjects showing lesions of various sizes and mixed composition [**(A)** (subject 042), **(B)** (subject 034), and **(C)** (subject 051)] and with primarily CSF [**(D)** (subject 055)]. Ethical approval was acquired to record and publish the MRI slices with consent from the participants (see NL58437.091.17).

A challenge of MRI-based volume conduction models in stroke patients is that (1) there is a large intersubject variability in lesion location and size (Wagner et al., 2007; Minjoli et al., 2017) and (2) the electric conductivity of the lesion is likely a commonly overlooked source of variability. Currently, most models with stroke lesions assume that the lesion consists only of cerebrospinal fluid (CSF) (Wagner et al., 2007; Datta et al., 2011; Minjoli et al., 2017), primarily based on 1-week post-stroke histology experiments in rodents (Jacobs et al., 2001; Soltanian-Zadeh et al., 2003). However, by visual inspection of MRI of chronic stroke patients, the composition of stroke lesions does not always appear as solely CSF (for examples from our patient sample, see Figure 1). Furthermore, a recent review showed that non-invasive measurements of lesion conductivity were highly variable, ranging from 0.1 to 1.77 S/m (McCann et al., 2019). Since simulation studies showed that the lesion conductivity could strongly affect the electric field generated by tDCS (Johnstone et al., 2021; Piastra et al., 2021a), knowing the lesion conductivity is vital in order to apply tDCS as intended.

Several methods have been proposed to estimate individualized head tissue conductivity (see McCann et al., 2019 for an overview). Among others, combined transcranial stimulation and scalp potentials have been used to estimate head tissue conductivity *in vivo* (Oostendorp et al., 2000; Gutiérrez et al., 2004; Dannhauer et al., 2011). A transcranial current is applied in these methods, and the induced scalp potentials are recorded using electroencephalography (EEG) electrodes. At the same time, a volume conductor model of the head is used to compute the scalp potentials assuming specific tissue conductivity. With the volume conductor model, the conductivity of one or more tissues can be estimated by varying the assumed tissue conductivity and minimizing the difference between the recorded and simulated potentials.

A combined transcranial stimulation-EEG-modeling approach has not yet been used for estimating stroke lesion conductivity. The goal of this simulation study is to demonstrate that simultaneous transcranial stimulation and EEG are suitable to estimate chronic stroke lesion conductivity in realistic experimental conditions.

## Materials and Methods

### Data Acquisition

T1-weighted MRI recordings were acquired from 12 chronic stroke subjects (all > 1 year post-stroke, see Table 1). All MRIs were recorded using a 3T MAGNETOM Prisma or 3T MAGNETOM PrismaFit scanner. The anonymized MRI scans are available online through the Donders Data Sharing Collection (Piastra et al., 2021b). All MRI data were acquired under the approval of the Ethics Committee “CMO regio Arnhem-Nijmegen” (NL58437.091.17) (Piastra et al., 2021b) with the written informed consent of all patients.

**TABLE 1:** Stroke lesion volume and optimal stimulation pairs to estimate the lesion conductivity for each subject.

**Subject**	**Lesion volume (ml)**	**Lesion depth (mm)**	**Anode**	**Cathode**
034	37.3	38.5	I2	FTT9h
035	11.9	35.0	TPP10h	Fp1
041	0.2	24.9	P9	FT10
042	13.1	40.5	T7	FTT10h
046	58.9	40.8	P10	F7
048	11.2	38.5	P10	TP7
050	0.1	38.2	P9	F8
051	48.9	37.7	P9	Fp2
053	0.3	25.2	I1	FT10
054	53.3	39.4	FTT10h	FTT9h
055	53.5	36.6	FT9	F8
056	85.2	35.8	TPP10h	TTP7h

### Volume Conductor Model

A four-compartment boundary element model was created from the MRI scan of 12 chronic stroke subjects, using the FieldTrip toolbox (Oostenveld et al., 2011). The models consisted of scalp, skull, CSF, and brain compartments, all modeled with 3,200 mesh elements. The lesion of each stroke patient was segmented using the LINDA algorithm (Pustina et al., 2016). The lesion volumes ranged from 0.1 to 85 ml. In order to assess the effects of lesion depth on the conductivity estimation, we calculated the depth of each lesion as the distance from the lesion centroid to the nearest node of the scalp compartment. For each patient, we created a model without and with the lesion.

MR images of subjects in our sample indicated that the lesion contained mainly CSF (1.71 S/m; McCann et al., 2019; Figure 1D), whereas other patients had clear signs of the presence of brain tissue (0.37 S/m) in the lesion (Figures 1A–C). Given this variation and the range described in the literature (0.1–1.77 S/m, McCann et al., 2019), we modeled the lesion consecutively with three conductivities: 0.74, 1.23, and 1.71 S/m. The conductivities assigned to scalp, skull, CSF, and brain were, respectively, 0.414, 0.016, 1.71, and 0.37 S/m.

The transcranial stimulation was simulated as described by Oostendorp et al. (2000): the stimulation electrodes were modeled as current monopoles and located 3 mm inside the scalp compartment. The scalp and skull surface meshes were refined near the stimulation electrodes to account for the large gradient of the electric potentials in that region, resulting in—for each patient—approximately 4,000 elements for the scalp and skull compartments. We used the boundary element method to compute the electric potential at the surface of the tissue compartments (Barnard et al., 1967; Oostendorp et al., 2000; Fuchs et al., 2002; Oostenveld and Oostendorp, 2002; Akalin-Acar and Gençer, 2004; Kybic et al., 2006; Stenroos and Sarvas, 2012; Makarov et al., 2020), as the result of a 0.1 mA stimulation current.

### Stimulation Configurations

To estimate the lesion conductivity from recorded potentials, the recorded potentials needed to be affected substantially by the presence of a lesion. Therefore, we identified the optimal stimulation electrode pair for each lesion model as the pair with the highest root-mean-square difference (RMSD) in scalp potentials between the same head model with and without a lesion. We performed this step for all patients separately to control for any between-subject differences in the lesion location and size, which cannot be achieved with fixed electrode montages. We considered from the 128 EEG electrodes in the international 10/5 system (Oostenveld and Praamstra, 2001) the subset of electrodes on the outer edge (Fp1/Fp2, F7/F8, FT9/FT10, FTT9h/FTT10h, T7/T8, TTP7h/TTP8h, TP7/TP8, TPP9h/TPP10h, P9/P10, and I1/I2) as potential stimulation electrodes. For each possible pair of these stimulation electrodes, the resulting scalp potentials were calculated at the remaining 126 electrodes not used for stimulation. These scalp potentials were then used to identify the optimal electrode pair based on the RMSD between the model with the lesion and the model without the lesion.

### Construction of Recorded Potentials

We simulated scalp potentials for the optimal stimulation pair and extracted data from either 8, 16, 32, 64, and 128 electrodes to investigate the quality of the conductivity estimation with an increasing number of electrodes. For the subset of eight electrodes, we used the eight electrodes closest to the Cz electrode (i.e., Cz, FCz, CPz, C1, C2, FFC1h, Fz, and AFF1). We included an additional electrode at the nasion as the reference electrode for the EEG recordings.

To reflect realistic experimental scenarios, we simulated electrode position errors by imposing a rotation of 0°–5° (corresponding to mean displacements of 0–9 mm, respectively) of the electrode positions around the coronal and sagittal head axes.

### Conductivity Estimation

The computed electrode potentials for the optimal stimulation pair were regarded as the measured potentials in an experimental setting, and we will refer to it as the “recorded” potential ψ.

The lesion conductivity was then estimated by the non-linear parameter estimation procedure described in Oostendorp et al. (2000). In this procedure, first, 10 random initial estimates ${\hat{\sigma }}_{0}$ for the lesion conductivity are chosen in an interval between 0.033 and 2 S/m, and the simulated electrode potentials $\varphi \,\left({\hat{\sigma }}_{0}\right)$ for every conductivity value are computed. Based on the difference between the “recorded” potentials ψ and the simulated model potentials $\varphi \,\left({\hat{\sigma }}_{0}\right)$, an improved estimate of the lesion conductivity ${\hat{\sigma }}_{1}$ is determined. This process is re-iterated until convergence is reached, defined as <0.1% change in the value of ${\hat{\sigma }}_{k-1}$ and ${\hat{\sigma }}_{k}$ at iteration *k*. We repeated this procedure for each combination of electrode numbers and position errors on the electrodes. Finally, we used the absolute error between the estimated conductivity ${\hat{\sigma }}_{k}$ and the actual conductivity used for the “recorded” potentials as a measure for the quality of the conductivity estimation.

## Results

**FIGURE 2:**
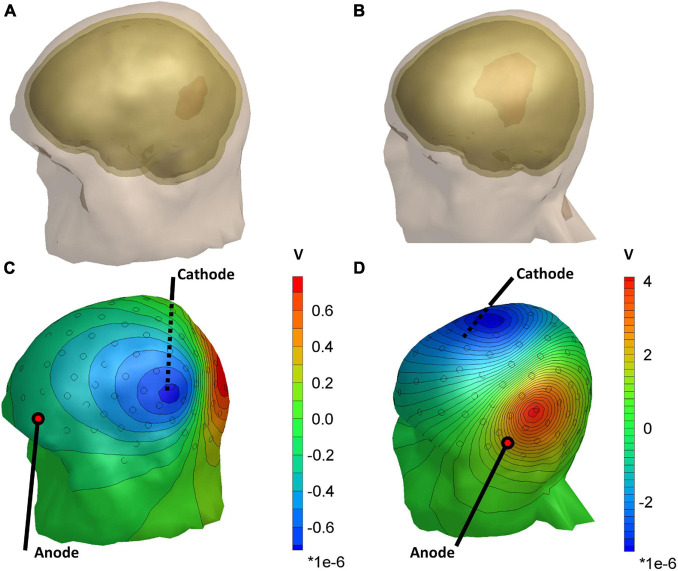
Head models of subject 035 **(A)** and subject 055 **(B)**, showing the lesion volume in red. **(C,D)** The distribution of the difference in scalp potentials between the models with and without the lesion and isopotential lines for the optimal stimulation pair. Black circles represent the 128 measurement electrodes. Note that the magnitude of the color bar varies between the two subjects.

Figure 2 shows the differences in scalp potentials between the models with and without the lesion for subject 035 (small lesion) and subject 055 (large lesion) for the optimal stimulation pair (for an overview of all subjects, see Table 1). For most subjects, the anodes of the optimal electrode pairs were primarily located around the left temporal area of the head and the cathodes around the right temporal area (see Supplementary Figure 1). However, subjects 034, 035, 051, and 053 had an electrode pair that consisted of frontal (Fp1/Fp2) or occipital (I1/I2) electrodes combined with a temporal electrode. The electric potential difference between the models with and without the lesion showed similar patterns for both subjects: positive potential differences in the vicinity of the anode and negative difference near the cathode. However, the effect of the larger lesion (subject 055, 53.5 ml) on the scalp potentials was about four times larger than for the smaller lesion (subject 035, 11.9 ml).

**FIGURE 3:**
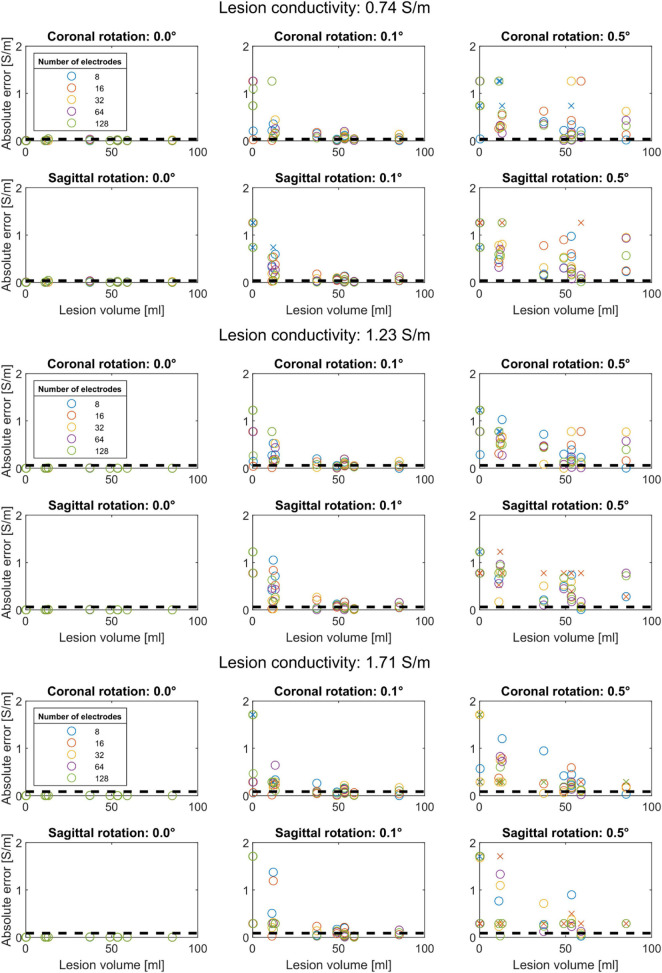
Conductivity estimation accuracy for coronal rotation (first row) and sagittal rotation (second row) for a lesion conductivity of 0.74, 1.23, and 1.71 S/m. Each color represents a different subset of electrodes. The black dashed lines indicate a 5% relative error to the modeled conductivity. Conductivity estimations that did not converge are marked with an “x”. Each panel shows that the absolute conductivity estimation error (*y*-axis) reduces with increasing lesion volume (*x*-axis) for rotations up to 0.5°. Without electrode rotation (left column), the conductivity is correctly estimated regardless of lesion size. At the lowest lesion conductivity (0.74 S/m), the estimation procedure is more sensitive to coronal and sagittal electrode rotation, as reflected by larger absolute errors, compared to higher lesion conductivity (1.23 and 1.71 S/m).

We found that the conductivity of all lesions was estimated correctly in the absence of electrode rotation (Figure 3). For the lesions with the lowest conductivity (0.74 S/m), rotation in coronal direction resulted in mean absolute errors of 0.12 ± 0.18 (mean ± sd) S/m for 0.1° and 0.24 ± 0.18 for 0.5° rotation. In the sagittal direction, absolute errors of 0.12 ± 0.15 and 0.43 ± 0.28 S/m were found for 0.1° and 0.5° rotation, respectively. Figure 3 also shows that the estimation errors were highly dependent on lesion size. However, lesion size alone could not fully explain the estimation errors. Lesions larger than 60 ml could be estimated with relative errors near 5%. Interestingly, the 48.9 and 58.9 ml lesions had lower estimation errors than the 53.2 and 85.2 ml lesions. These differences also did not seem to be related to lesion depth, as the smaller lesions were located deeper inside the brain (40.8 and 37.7 mm compared to 35.8–39.4 mm).

For both coronal and sagittal electrode rotation, the estimation error increased with increasing rotation angles, regardless of the lesion size. However, the estimation error did not appear to be consistently related to the number of electrodes used for the conductivity estimation. For instance, for 0.72 S/m lesions and 0.1° coronal rotation, the 11.2 ml lesion (subject 048) was estimated with an error of 0.01 S/m using 16 electrodes. However, the absolute error ranged from 0.06 to 0.17 S/m for the other electrode subsets. Furthermore, for 0.1° coronal rotation, the 37 ml lesion was estimated with an absolute error of at least 0.06 S/m. However, when rotated 0.1° in the sagittal direction, the 37 ml lesion was estimated with absolute errors below 0.05 S/m. For rotation up to 0.5° in coronal direction, the conductivity of lesions larger than 48 ml was estimated with errors below 0.05 S/m for 64 and 128 electrode subsets. In contrast, an opposite pattern was observed for rotation in the sagittal direction: increasing the rotation to 0.5° resulted in estimation errors ranging up as high as the lesion conductivity itself, indicating high sensitivity for coronal rotation.

The effect of modeled lesion conductivity was also tested for all models and electrode rotations. The robustness to 0.5° rotation improved for higher lesion conductivity, with similar mean absolute errors but relative errors reducing from 0.32 ± 0.24 for 0.74 S/m lesions to 0.21 ± 0.17 for 1.72 S/m lesions. For rotations above 1° in either coronal or sagittal direction, the optimization algorithm never converged to the correct lesion conductivity for any combination of electrode subset, lesion volume, or lesion conductivity.

## Discussion

We propose a method that combines MRI, EEG, and transcranial stimulation to estimate the conductivity of cortical stroke lesions experimentally. We simulated this method in head models of 12 chronic stroke patients with lesion volumes within the ranges reported in the literature (Chen et al., 2000) and evaluated the effect of the number of EEG recording electrodes and errors in EEG electrode placement. We found that the optimization algorithm converged to the correct lesion conductivity value when noise on the electrode positions was absent. In the case of electrode rotations, estimation error depended on lesion size. However, the conductivity of lesions larger than 50 ml could be estimated with low relative errors when coronal and sagittal rotations remained at 0.1°.

The method we propose requires only a single post-stroke MRI to estimate the lesion conductivity. In the first step of our method, we identified the optimal stimulation pair to estimate the lesion conductivity by evaluating the RMSD between a model with and without the lesion. We found optimal electrode pairs that were localized mainly around the left and right temporal areas. Likely, this is a consequence of the used patient sample, which consisted of stroke patients with lesions in approximately the same regions. Therefore, the optimal stimulation electrode pair is expected to be more variable for lesions at different locations.

The accuracy of the conductivity estimation method depends on several factors. For instance, the accuracy depended on lesion volume; larger lesions more strongly affect scalp potentials than smaller lesions. However, we observed some inconsistency in this pattern, which could not be explained by our measure for the lesion’s depth. Nonetheless, more superficial lesions are expected to have a more profound effect on scalp potentials than lesions located deeper inside the brain. However, the measure we used for lesion depth—the distance between the lesion’s centroid and the nearest scalp node—might not have been able to take this effect into account when calculated independently of the lesion’s size.

Another explanation for the observed differences in estimation accuracy could be that the effects of small lesions were not sufficiently captured by the subsets of electrodes we used. For instance, Figures 2C,D show that the lesion introduces only local electric potential differences at the scalp. At the same time, the subsets of 16–128 electrodes we used were distributed uniformly over the scalp. Likewise, the subset of eight electrodes around Cz could be suboptimal if it does not record the largest potential differences due to the lesion. Therefore, selecting a subset of electrodes including only the most affected electrodes—which would vary per subject—could improve our proposed method for small lesions.

An additional factor influencing the accuracy of our results is the lesion conductivity we assumed in the models. We modeled the lesion with three different conductivity values, in-between two times the modeled brain conductivity and CSF conductivity. Like lesion volume, higher lesion conductivity increases the effect the lesion has on the scalp potentials. This is confirmed by the lower absolute errors we found for increased conductivity. Furthermore, the method proved more robust to electrode rotations for lesions with higher conductivity.

Although the conductivity of larger lesions (>50 ml) could successfully be estimated, we found that the conductivity estimation procedure is sensitive to incorrect electrode positions. Especially, rotation in the sagittal direction was detrimental to the conductivity estimation accuracy, which may be explained by the orientation of the isopotential lines near the electrodes that record the strongest effect of the presence of the lesion (Figures 2C,D). For coronal rotation, the electrodes rotate more tangent to the isopotential lines, resulting in lower relative differences between the recorded and modeled scalp potentials. This hypothesis is in line with the relatively high robustness to the sagittal rotation of the 37.2 ml lesion of subject 034, for whom an optimal stimulation pair consisting of I2 and FTT9h was found.

For electrode rotations above 1°, the optimization algorithm did not correctly estimate the lesion conductivity. In this situation, scalp potential differences due to electrode position errors surpass those introduced by the lesion. As a consequence, the optimization algorithm can only minimize these errors with unrealistic lesion conductivities, resulting in high relative errors. However, it should be noted that systematic rotations represent a worst-case scenario: in experimental conditions, electrode placement errors may be distributed randomly. Nonetheless, the estimation method results suggest that mean recording electrode position errors should remain below 0.1° (1 mm mean displacement) to keep estimation errors below 5%. These accuracies can only be realized with 3D scanning techniques (Dalal et al., 2014). When applying this method in practice, the patients should ideally wear an MRI-compatible EEG cap during the MRI acquisition to minimize the co-registration error and maximize the conductivity estimation accuracy.

Future work comprises the estimation of the range of lesion conductivities in stroke patients. Furthermore, the effect of more realistic volume conductor models with a more realistic description of the brain, i.e., a separate gray matter and white matter volume, remains to be explored.

### Limitations

We did not add random noise reflecting background EEG activity to the scalp potentials. The effect of random noise can be compensated for by either averaging over a prolonged stimulation time or increasing the stimulation intensity. At this point, we simulated stimulation at an intensity of 0.1 mA, which ensures that the method can be applied with low discomfort to the patient. Also, we did not fully control for the depth of the lesions. The conductivity of lesions distant from the scalp, i.e., subcortical lesions, will be more challenging to estimate and potentially explain the inconsistency in the relation between lesion size and conductivity estimation error we observed. However, considering lesion size and depth as independent measures may be an oversimplification that did not explain the inconsistency between lesion size and the observed conductivity estimation error.

We used a four-compartment model without a separate representation of gray and white matter. This simplification was made to reduce the computational load that the segmentation of the complex structure of the brain would introduce. As an alternative, the finite element method would be a more suitable approach to model the human head more efficiently and realistically. The modeled conductivities for the scalp, skull CSF, and brain were based on the literature (McCann et al., 2019) and assumed known. However, skull conductivity varies significantly between individuals (McCann et al., 2019), and an inaccurate assumption would translate to low accuracy of the lesion conductivity estimation. One potential solution is to estimate the skull conductivity based on the scalp potentials in electrodes whose potentials are affected minimally by the lesion.

## Conclusion

In conclusion, estimating the lesion conductivity can easily be incorporated in experimental procedures that combine tDCS, EEG, and MRI for individualized head models. The achievable estimation accuracy depends on the balance between lesion volume, lesion depth, lesion conductivity, and the measurement electrodes’ co-registration error. The accuracy of MRI-based volume conductor models can be improved by including an individualized estimate of the stroke lesion conductivity with our proposed method. As a result, this can lead to the improved application of transcranial electric stimulation in stroke patients.

## Data Availability Statement

Publicly available datasets were analyzed in this study. These data can be found here: https://data.donders.ru.nl/collections/di/dcmn/DSC_4020000.14_955?0.

## Ethics Statement

The studies involving human participants were reviewed and approved by the CMO Regio Arnhem-Nijmegen. The patients/participants provided their written informed consent to participate in this study.

## Author Contributions

JC performed the data analysis. All authors were involved in the design of the study, interpretation of the results, drafted and revised the manuscript, and read and approved the final manuscript.

## Conflict of Interest

The authors declare that the research was conducted in the absence of any commercial or financial relationships that could be construed as a potential conflict of interest.

## Publisher’s Note

All claims expressed in this article are solely those of the authors and do not necessarily represent those of their affiliated organizations, or those of the publisher, the editors and the reviewers. Any product that may be evaluated in this article, or claim that may be made by its manufacturer, is not guaranteed or endorsed by the publisher.
