# Supplementary material for: A Method to Experimentally Estimate the Conductivity of Chronic Stroke Lesions: A Tool to Individualize Transcranial Electric Stimulation
Source: Front Hum Neurosci. 2021 Oct 12;15:738200. doi: 10.3389/fnhum.2021.738200 (PMC8546262; doi:10.3389/fnhum.2021.738200)
Supplement: Supplementary file 1 [file Data_Sheet_1.DOCX]

**Supplementary Figures**


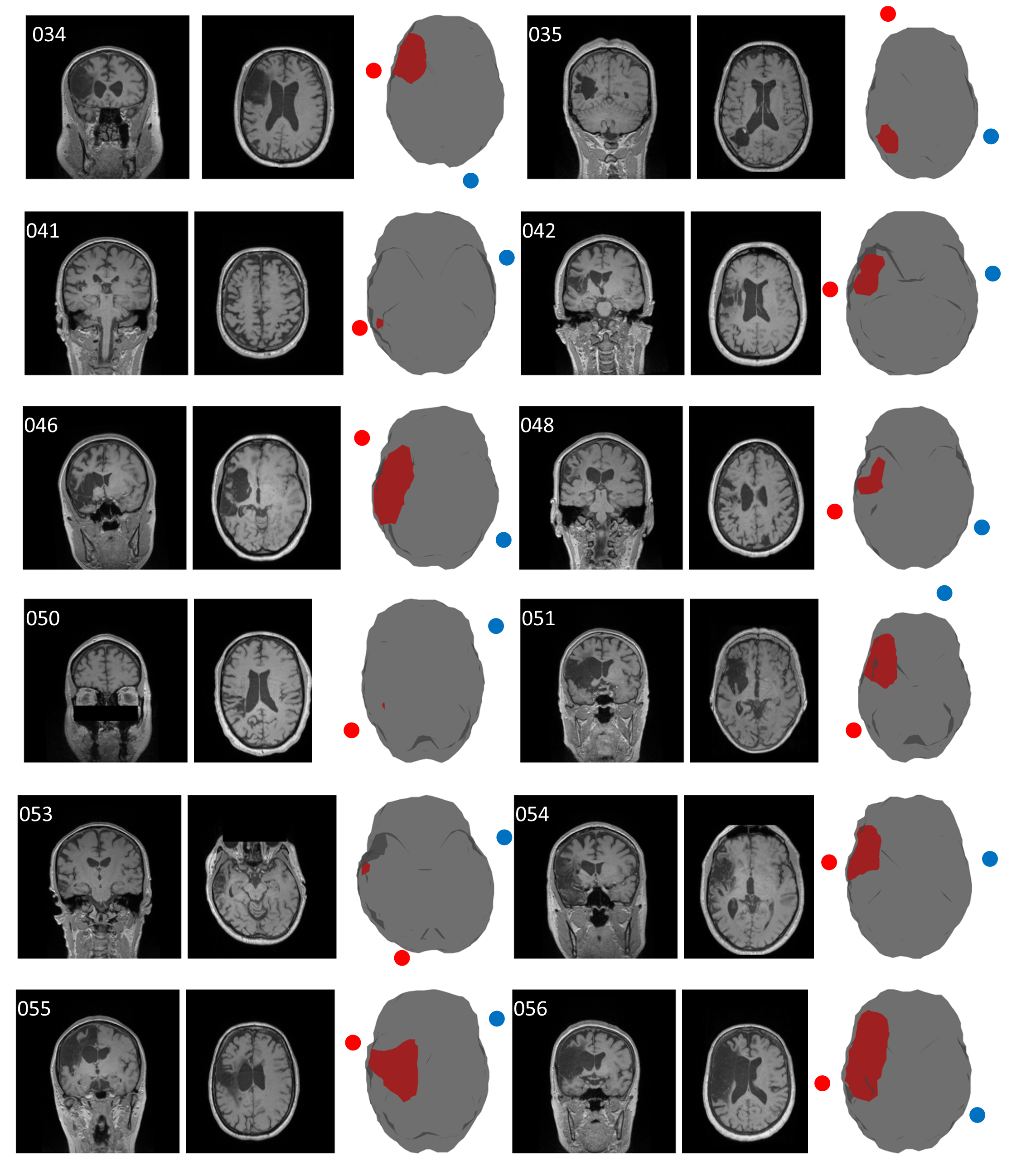


**Supplementary Figure 1. Two MRI slices across the lesion and the brain (grey) and lesion (red) models for each subject, top view. The markers in the model plots indicate the anode (red) and cathode (blue) of the optimal stimulation pair.**
